# Structural Basis for Broad Substrate Selectivity of Alcohol Dehydrogenase YjgB from *Escherichia coli*

**DOI:** 10.3390/molecules25102404

**Published:** 2020-05-21

**Authors:** Giang Thu Nguyen, Yeon-Gil Kim, Jae-Woo Ahn, Jeong Ho Chang

**Affiliations:** 1 Department of Biology Education, Kyungpook National University, 80 Daehak-ro, Buk-gu, Daegu 41566, Korea; thugiang1995@gmail.com; 2 Beamline Science Division, Pohang Accelerator Laboratory, 127 Jigok-ro, Nam-Gu, Pohang, Gyoungbuk 37673, Korea; ygkim76@postech.ac.kr; 3 Postech Biotech Center, Pohang University of Science and Technology, 77 Cheongam-ro, Nam-Gu, Pohang, Gyoungbuk 37673, Korea; 4 Department of Biomedical Convergence Science and Technology, Kyungpook National University, 80 Daehak-ro, Buk-gu, Daegu 41566, Korea

**Keywords:** YjgB, aldehyde dehydrogenase, broad specificity, crystal structure, hydrophobic pocket, docking-simulation

## Abstract

In metabolic engineering and synthetic biology fields, there have been efforts to produce variable bioalcohol fuels, such as isobutanol and 2-phenylethanol, in order to meet industrial demands. YjgB is an aldehyde dehydrogenase from Escherichia coli that shows nicotinamide adenine dinucleotide phosphate (NADP)-dependent broad selectivity for aldehyde derivatives with an aromatic ring or small aliphatic chain. This could contribute to the design of industrial synthetic pathways. We determined the crystal structures of YjgB for both its apo-form and NADP-complexed form at resolutions of 1.55 and 2.00 Å, respectively, in order to understand the mechanism of broad substrate selectivity. The hydrophobic pocket of the active site and the nicotinamide ring of NADP(H) are both involved in conferring its broad specificity toward aldehyde substrates. In addition, based on docking-simulation data, we inferred that π–π stacking between substrates and aromatic side chains might play a crucial role in recognizing substrates. Our structural analysis of YjgB might provide insights into establishing frameworks to understand its broad substrate specificity and develop engineered enzymes for industrial biofuel synthesis.

## 1. Introduction

The production of large yields of bio-renewable chemicals and biofuels from biomass is a primary goal in the fields of metabolic engineering and synthetic biology. The production of butanol, isobutanol, propanediol, butanediol, and aromatic alcohols while using designed synthetic pathways of microorganisms has been attempted to meet industrial demands [1,2,3,4,5,6]. In the synthetic pathway of these alcohols, the final step is commonly the reduction of an aldehyde through alcohol dehydrogenases. *Escherichia coli* possess strong alcohol dehydrogenases that could contribute to producing alcohols through a designed pathway [7]. These powerful enzymes, including YjgB, YahK, and YqhD, show nicotinamide adenine dinucleotide phosphate (NADP)-dependent activities for a variety of aldehydes. YqhD has 17% sequence identity with YjgB and it shows reductase activity for broad short-chain aldehydes, including butyraldehyde, for which it shows the strongest activity, as well as glyceraldehyde, malondialdehyde, isobutyraldehyde, methylglyoxal, propanealdehyde, acrolein, furfural, glyoxal, 3-hydroxypropionaldehyde, glycolaldehyde, acetaldehyde, and acetol [8]. Unlike YqhD, YjgB, and YahK, which have a similarity of 35% sequence identity, strongly prefer benzaldehyde and furfural. In terms of turnover number, hexanal is supposed to be the best substrate for both enzymes. However, no activity has been measured when using a ketone as a substrate [7]. YjgB and YahK have been utilized in pathway design for the production of aromatic compounds for 2-(4-hydroxyphenyl)ethanol and 4-hydroxyphenyllactic acid. In addition, YjgB has recently been reported to produce 2-phenylethanol (2-PE) and 2-phenylethyacetate (2-PEAc) in *E. coli*-based metabolic engineering [9,10]. These 2-PE and 2-PEAc are fragrance compounds containing high-value flavor with a wide range of applications in the cosmetic, perfumery, and food industries. Although the chemical synthesis of 2-PE and 2-PEAc has advantage in cost, many consumers prefer more natural or bio-products in the industry of flavor [10,11]. On the other hand, natural extracts obtained from plant sources have high-cost, due to their low extraction yields [12,13].

Structurally, YjgB, together with YahK and YqhD, are grouped in the medium-chain dehydrogenase/reductase superfamily. YqhD belongs to the family III metal-dependent polyol dehydrogenases, while YjgB and YahK are classified as members of the cinnamyl alcohol dehydrogenase family [14,15]. Generally, several medium-chain alcohol dehydrogenases have been revealed to exist in bacteria as dimers or tetramers, while using NAD(H) or NADP(H) as a cofactor and one catalytic zinc ion at the active site. In some dimeric alcohol dehydrogenases, an additional zinc might be required to further support the stability of the external loop structures [16]. Like other Zn-containing members in the family, YjgB and YahK both possess the GHEX_2_GX_5_(G,A)X_2_(I,V,A,C,S) motif and the GX_1–3_GX_1–3_G pattern located in the nucleotide-binding region [7]. Although Sulzenbacher et al. comprehensively studied the YqhD structure [17], little information is available regarding the other two versatile enzymes, except for the YahK structure, which is deposited in the Protein Data Bank (PDB code: 1UUF) without an attached report. To fill this gap, this study aims to determine the YjgB apo and complex structures of *E. coli* and compare them with the structures of YahK and YqhD to indicate the substrate specificity of YjgB. The findings of this study are useful to the field of protein engineering, as they inform the better production of 2-PE and 2-PEAc.

## 2. Results

### 2.1. Overall Structure

**Figure 1:**
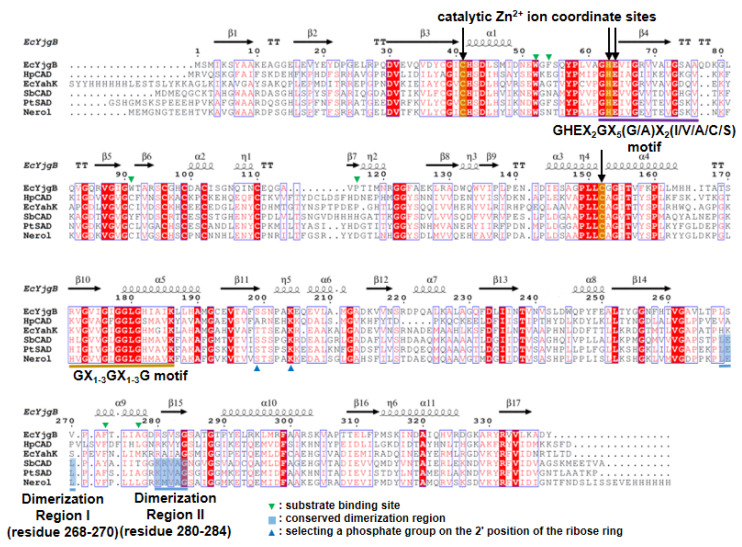
Sequence alignment for *Ec*YjgB, *Hp*CAD, *Ec*YahK, *Sb*CAD, *Pt*CAD, and Nerol. Sequences of *Ec*YjgB, *Escherichia coli* BL21 (DE3) YjgB (B21_04099); *Hp*CAD, *Helicobacter pylori* cinnamyl alcohol dehydrogenase (PDB code: 3TWO); *Ec*YakK, *Escherichia coli* BL21 (DE3) YahK (PDB code: 1UUF); *Sb*CAD, *Sorghum bicolor* cinnamyl alcohol dehydrogenase (SbCAD) (PDB code: 5VKT); *Pt*SAD, *Populus tremuloides* SAD (sinapyl alcohol dehydrogenase) (PDB code: 1YQD); Nerol, *Yersinia pseudotubercluosis* IP32953 nerol dehydrogenase (PDB code: 5Z0C) are aligned. Sequence identities for *Ec*YjgB are 30.4% (*Hp*CAD), 35.9% (*Ec*YakK), 35.0% (*Sb*CAD), 35.9% (*Pt*SAD), and 34.7% (Nerol), respectively. Down arrows, and purple and golden under-bars indicate catalytic Zn^2+^ ion coordinate sites, GHEX_2_GX_5_(G/A)X_2_(I/V/A/C/S), and GX_1–3_GX_1–3_G motif, respectively. Green downside and blue upside triangles indicate substrate-binding sites and the amino acids important for selecting the phosphate group on the 2′ position of the ribose ring in nicotinamide adenine dinucleotide phosphate (NADP). Sky blue boxes represent conserved dimerization region. This multiple sequence alignment was performed using *Clustal/W* and prepared with the *ESPript* software package [20,21].

**Figure 2:**
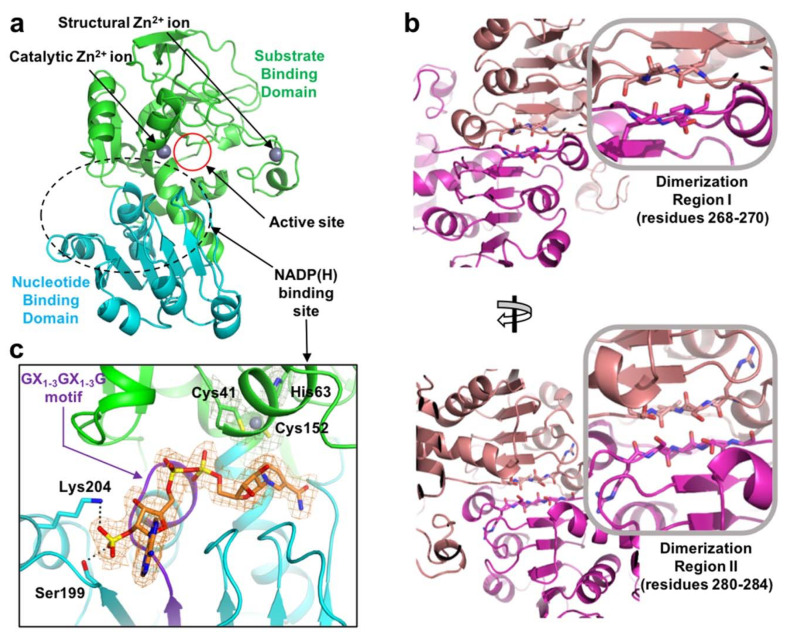
Structure of *Escherichia coli* YjgB (*Ec*YjgB). (**a**) Overall structure. (**b**) Dimeric interface of *Ec*YjgB. In the outcome of the Proteins, Interfaces, Structures, and Assemblies (PISA) analysis, the dimeric chains are colored as salmon and purple [19] (**c**) NADP binding site. The SBD (substrate-binding domain) and NBD (nucleotide-binding domain) are represented as green and cyan, respectively. The *2Fo-Fc* electron densities of NADP (orange), and zinc ion (grey) with its coordinated residues (green) are shown at the 1.0 σ contoured level with their corresponded colors. The GX_1–3_GX_1–3_G motif is indicated as a purple cartoon.

The *E. coli* YjgB (*Ec*YjgB) was crystallized in the monoclinic space group, *C2*, and its crystal structure was successfully determined at 1.55 Å resolution (Table 1). As predicted from sequence alignment (Figure 1), the *Ec*YjgB structure was found to belong to a typical cinnamyl alcohol dehydrogenase (CAD) family [17] and sinapyl alcohol dehydrogenase (SAD) family [17], which consists of a nucleotide binding domain (NBD, residues 165–286) and a substrate-binding domain (SBD, residues 1–164 and 287–338) (Figure 2a). The NBD (nucleotide-binding domain) was shown to adopt a canonical Rossmann fold [18] that was composed of alternating beta strands and alpha helixes, which can hold cofactor NAD(H)/NADP(H). As in other Zn-dependent CAD and SAD families, PDBePISA (Proteins, Interfaces, Structures, and Assemblies) analysis showed that this Rossmann fold can form a dimer [19]. The structure of the dimeric interface showed that dimerization region I (residues 268–270) and II (residues 280–284) establish the dimerization interface by generating two β-sheets (Figure 2b). These dimerizations are involved in forming a hydrophobic active site that consists of residues with aromatic rings (see 2.2 for more detail).

As the GX_1–3_GX_1–3_G, GHEX_2_GX_5_(G/A)X_2_(I/V/A/C/S) motif, and catalytic Zn^2+^ ion coordination sites, Cys41, His63, and Cys152, are conserved in sequence alignment (Figure 1), they play a role in coordinating Zn^2+^ ions and generating a NAD/NADP binding site. The electron density of NADP is clearly shown in the NADP(H) binding site of the *Ec*YjgB-NADP complex structure (Figure 2c). We also found that the NADP was well overlaid with NADP(H) molecules of CAD/SAD structures (data not shown). We observed that His42, Ser43, Ser46, Gly179, Leu180, Ser199, Ser200, Asn201, Glu208, Ser285, Gly327, and Arg332 constitute the NADP(H) binding site. In addition, conserved Ser199 and Lys204 (Figure 2c) make it possible to specifically select the 2′ phosphate of NADP(H) as a stabilizing 2′ phosphate of the dinucleotide while using a hydrogen bond and charge-charge interaction.

### 2.2. Zinc Coordination and Active Site

**Figure 3:**
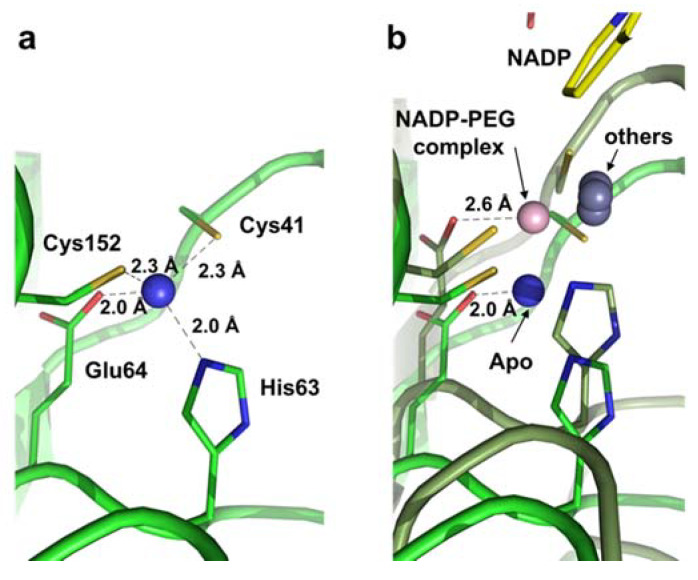
Catalytic zinc coordination. (**a**) Catalytic zinc coordination and its coordinated residues in the apo structure. The blue sphere indicates a zinc ion of *Ec*YjgB. (**b**) Catalytic zinc coordination of superposed apo and NADP complex structures. The blue and light-pink spheres indicate the catalytic zinc ions of apo and NADP complex, respectively. The apo and NADP complex structures are drawn by green and dark-green cartoons, respectively.

We observed a cleft between the SBD (substrate-binding domain) and NBD (nucleotide-binding domain) that forms the active site with a catalytic Zn^2+^ ion (Figure 2a). It was found that the catalytic Zn^2+^ ion is coordinated by the tetrahedral combination of Cys41, His63, Glu64, and Cys152, which were well conserved (Figure 1). In the apo structure, distances ranging from 2.0 to 2.3 Å were shown between the zinc ion and its coordinated resides; Cys41 (2.3 Å), His63 (2.0 Å), Glu64 (2.0 Å), and Cys152 (2.3 Å) (Figure 3a). Meanwhile, each position of the four residues was 0.6 Å farther away from catalytic zinc in the *Ec*YjgB-NADP complex, based on its superimposed structure with the apo form through the NBD region (Figure 3b). Although the catalytic components were relatively relocated toward the nicotinamide ring of NADP, their positions were still not properly matched to those of other CAD/SAD structures.

Interestingly, in the *Ec*YjgB-NADP complex structure, we found an electron density of polyethylene glycol (PEG) density in the vicinity of the active site (see Section 2.3). It seemed that PEG did not induce the zinc relocation by dissociation of the glutamate residue since PEG does not contain an aldehyde group, as observed in the structures of *Sb*CAD (PDB code: 5VKT) [17], yeast alcohol dehydrogenase (PDB code: 4W6Z) [22], and *E.coli* ethanol inducible dehydrogenase (PDB code: 4GKV) [23]. Consequently, the catalytic zinc could not go further toward a substrate as well as generate a zinc-bound alkoxide ion, which might cause insufficient relocation of catalytic zinc in the *Ec*YjgB-NADP complex structure.

### 2.3. Substrate-Binding Pocket by Conformational Change

**Figure 4:**
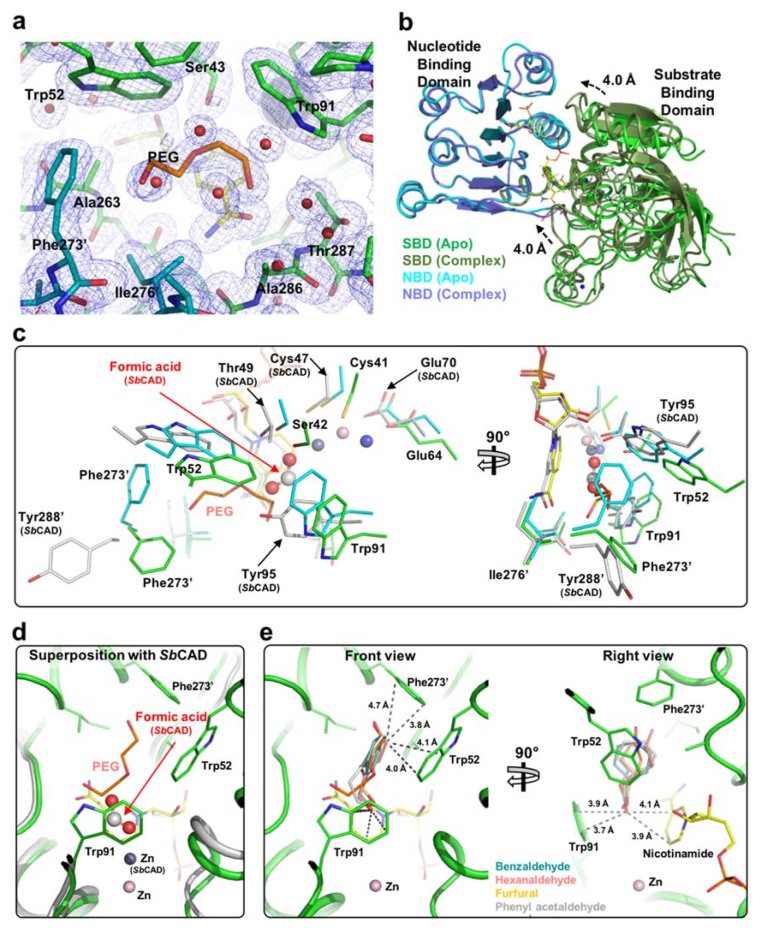
Active site and Docking simulation. (**a**) Electron density map of the active site in the *Ec*YjgB-NADP-polyethylene glycol (PEG) complex structure. The *2Fo-Fc* densities including PEG are shown at the 1.0 σ contoured level with a dark-blue colored mesh. The monomer and its symmetry related molecule are colored in green and cyan, respectively. (**b**) Conformational difference between the apo and complex structure. The SBD (substrate-binding domain) of the apo and complex structures and NBD (nucleotide-binding domain) of the apo and complex structures are colored as green, dark green, cyan, and light purple, respectively. (**c**) Conformational change in the active site. Apo, NADP complex, and closed form of *Sb*CAD (PDB code: 5VKT) are colored in green, cyan, and gray colors, respectively. Formic acid in *Sb*CAD is represented as a ball and stick with gray-colored carbon and red-colored oxygen. Zinc ions of apo-YjgB, *Ec*YjgB-NADP, and closed form of *Sb*CAD are represented as light-blue, light-pink, and silver spheres, respectively. PEG in the *Ec*YjgB-NADP structure are drawn as stick with orange carbon and red oxygen. (**d**) Docking-simulation and comparison with PEG and formic acid of *Sb*CAD. *Ec*YjgB-NADP structure and *Sb*CAD are colored in green and silver, respectively. Formic acid in *Sb*CAD is represented as corresponded to Figure 4c. Zinc ions of *Ec*YjgB-NADP, and closed form of *Sb*CAD are shown in light-pink and gray spheres, respectively. PEG in the *Ec*YjgB-NADP structure are drawn as stick with orange-carbon and red-oxygen. (**e**) Two side views of active site in the *Ec*YjgB-NADP complex structure. Overlaid ligands calculated from the docking simulation are shown as indicated colors. Dashed lines represent the acceptable distances in π-π stacking. Light-pink sphere indicates the catalytic zinc of *Ec*YjgB-NADP complex.

We tried to obtain the crystal structure with the substrate or NADP to investigate the reaction mechanism of *Ec*YjgB for broad substrates. During the determination of the structure comprising NADP, we found that a polyethylene glycol (PEG) fragment occupied its catalytic cavity, which was quite similar to a hexanaldehyde (Figure 4a). As it is holding the NADP, the SBD moves about 4.0 Å toward the NBD with a root-mean-square deviation (r.m.s.d.) of 1.02 Å when compared to the apo structure (Figure 4b). It represents that NADP binding can generate the closed form, as observed in other CAD/SAD family. The superposition of the *Ec*YjgB-NADP complex structure with a closed form of *Sb*CAD implied that NADP binding is likely to induce the conformational change upon substrate-binding to hold a substrate or a PEG fragment (Figure 4c). Thus, several conserved catalytic residues were relocated to the vicinity of their counterpart in *Sb*CAD, such as Ser42, Trp52, Trp91, and Tyr95. The tryptophan residues may play a crucial role in holding PEG due to their aromatic rings. The catalytic zinc of NADP complex was not completely moved to the proper position, although PEG was occupied at the substrate-binding pocket, as mentioned previously. This might indicate that the absence of aldehyde group in PEG led positions of Cys41, Ser42, and Glu64 adjacent to those of counterparts in *Sb*CAD. Interestingly, Phe273′ of dimeric chain was located unalike to the closed form of *Sb*CAD (Figure 4c). In contrast, Tyr288′ faced outside of the substrate-binding pocket without an aromatic ring or its equivalent to that in the formic acid. Phe273′ turns toward the substrate binding site since PEG occupies the substrate-binding pocket after the conformational change upon NADP binding. Consequently, the oxygen from PEG nearby Phe273′ could form a kind of anion–π interaction that contributes to holding the ligand.

### 2.4. Substrate Specificity

The PEG occupancy could present the substrate-binding site reasonable and acceptable. The conformational change and the dimerization result in the substrate-binding site. The substrate-binding site was found to consist of Ser43, Trp52, Trp91, Thr287 from NBD, Ala263 and Ala286 of SBD, Phe273′, and Ile276′ from the other dimeric chain (Figure 4a). The combination of Trp52, Trp91, Phe273′, and Ile276′ was observed to constitute the analog of the hydrophobic cavity with appropriate hydrophobicity, polarity, and size for the substrate. Trp52 and Trp91 were shown to hold the ligand through CH-π stacking, which appeared to be a primary contributor for substrate recognition. Particularly, turning Phe273′ toward the substrate-binding pocket possibly forms a kind of anion-π interaction. Catalytic zinc-coordinating residues are similarly positioned to the closed form, despite their insufficient zinc location. Thus, we determined that the conformation of PEG and its interactions with catalytic residues are largely similar to those of authentic substrate.

We carried out docking-simulations while using *Autodock Vina* software in order to suggest the substrate-binding mode and critical residue for substrate binding [24]. A *Ec*YjgB protein that was derived from the *Ec*YjgB-NADP-PEG complex was used as a target molecule, because it could tightly bind substrates in the active site, which was constituted by proper arrangement of NADP and PEG. Hexanaldehyde, benzaldehyde, furfural, and phenylacetaldehyde, known as aldehyde substrates, were used for the docking simulation [7,9,10]. The highest calculated binding affinities were −5.7, −4.6, −4.3, and −6.1, respectively, for benzaldehyde, hexanaldehyde, furfural, and phenylacetaldehyde. Their calculated binding modes were somewhat comparable to the formic acid of *Sb*CAD as well as the PEG fragment of the NADP complex structure (Figure 4d). From the predicted binding mode, we could figure out the Trp52 function to hold a substrate with an aromatic ring or CH chain due to π-π stacking interactions. Trp52 is not perfectly stacked to substrates and it is found Parallel-Displaced π-π stacking to aromatic rings and CH chains, at distance of below ~4.1 Å (Figure 4e). In addition, Phe273′ of the dimeric chain seems to limit the size of substrates by forming T-shaped π-π stacking, despite not being complete vertically with distances of 3.8–4.7 Å. Finally, the oxygen of aldehyde from substrates are located between Trp91 and the nicotinamide ring of NADP (Figure 4e). They are regarded as perpendicular anion-π stackings with the distances of 3.7–4.1 Å. Therefore, it is acceptable to infer that these π-π stackings by aromatic side chain in active site play a critical role in holding substrates and conferring the broad selectivity.

## 3. Discussion

The final process is the reduction of an aldehyde. *E. coli* has strong aldehyde dehydrogenases with a broad specificity to bio-synthesize small size alcohols, such as butanol, isobutanol, propanediol, butanediol, 2-PE, and 2-PEAc. We determined the structure of YjgB from *E. coli* to understand this broad specificity and contribute to designing more efficient pathways for alcohol production. From this structure, we suggested the mechanism of broad substrate specificity. The active site was found to be constituted by the combination of isoleucine, alanine, tryptophan, phenylalanine, and the nicotinamide ring of NADP, which enable the broad specificity for small hydrophobic aldehydes with aromatic rings and short CH chains. This broad specificity of aldehyde dehydrogenases in *E. coli* might assist cell survival under oxidative stress [7]. It is known that aldehydes are very reactive and toxic to cells, since the aldehyde group (CHO) is a strong electrophile that can undergo additional reactions. Small molecular aldehydes are naturally generated in cells and produced in small amounts through the intermediary metabolism of natural chemicals. These aldehydes are primarily metabolized by dehydrogenases or glutathione-dependent pathways, and toxicity results from increased intracellular concentrations following these detoxification systems being overwhelmed [25]. Bacteria also encounter oxidative stresses: reactive oxygen species (ROS) are generated from a number of sources, including the leakage of single electrons from respiratory enzymes and environmental stresses, such as UV radiation [26]. Broad specificity helps the resistance of *E. coli* to small aldehydes with aromatic rings and CH chains. This broadness appears to confer more competitiveness and flexibility for a limited gene pool of bacteria.

In comparison of the substrate docking-simulation data to the NADP complex structure incorporating a PEG fragment (Figure 4d,e), the aldehyde groups on the substrates that are located toward the catalytic zinc are in opposition to the aldehyde out-group of the PEG complex structure. Despite the proper directions of aldehyde groups, their distances to the catalytic zinc are still insufficient to make zinc-ligated aldehyde intermediates. It is assumed that those outputs are caused by unreflecting complete induced fit in the target molecule during the docking-simulation. Therefore, the binding of an authentic substrate, not a PEG molecule, to the active site pocket leads to the formation of the activated zinc coordination and actual substrate bound conformation for catalytic reactions. The B-factors of zinc and Glu64 increased more than twice as Glu64 moved away from the zinc ion by NADP binding (Table 2). Given these, we can speculate that a substrate binds to the widened gap between Trp91 and nicotinamide ring, and the substrate can be likely located a little bit below the site of simulation. Regarding the zinc relocation that can be induced by the substrate-entry, it is expected that the zinc ion can approach the aldehyde group of the substrate to generate a zinc-bound substrate intermediate. Taken together, the NADP-PEG complex structure with multiple substrate docking simulations could contribute to understanding the substrate-binding mechanism in *Ec*YjgB.

## 4. Materials and Methods

### 4.1. Cloning, Expression, and Purification

The *YjgB* gene was digested with *Nde*I and *Xho*I restriction enzymes and inserted into a pET30a vector (Novagen) after PCR amplification. The *Ec*YjgB protein, containing the 6X His tag at its C-terminus, was expressed in the *E. coli* strain BL21 (DE3). The cells were cultured in an LB medium containing kanamycin at 37 °C until reaching an absorbance of 0.7 at 600 nm. Protein expression was induced by 1.0 mM isopropyl β-D-1-thiogalactopyranoside (IPTG), and the cells were then incubated for 18 h at 20 °C. After harvest, the cell pellets were resuspended in buffer A (40 mM Tris-HCl, pH 8.0, and 5 mM β-mercaptoethanol) and then disrupted by ultrasonication. The cell debris was removed by centrifugation at 12,000× *g* for 20 min., and the lysate was bound to Ni-NTA agarose (QIAGEN). After first washing with buffer A containing 20 mM imidazole, the bound proteins were then eluted with buffer A containing 300 mM imidazole. The *Ec*YjgB protein was purified while using size exclusion chromatography to obtain high purity for crystallization (Superdex200, GE Healthcare, Chicago, IL, USA). The purified proteins were concentrated to 20 mg/mL in a solution with 20 mM Tris-HCl, pH 7.5, and 5 mM β-mercaptoethanol.

### 4.2. Crystallization

Crystallization of the apo *Ec*YjgB was initially performed using crystal screening kits (Hampton Research Co., Aliso Viejo, CA, USA and Emerald Biostructures Co., Bainbridge Island, WA, USA) and by using the sitting-drop vapor-diffusion method at 20 °C. Each experiment consisted of mixing 1.0 μL of the protein solution with 1.0 μL of reservoir solution and equilibrating this against 0.1 mL of the reservoir solution. Thin plate-shaped crystals were observed after 1–3 days. The diffraction of these crystals was not suitable for the determination of their structure. We adjusted the concentration of the precipitant, polyethylene glycol (PEG) 3350, and the pH of the reservoir solution to obtain high purity for crystallization. The suitable crystals for diffraction were obtained under conditions of 0.1 M Tris-HCl, pH 7.0, 16% (*w*/*v*) PEG 3350, and 0.2 M lithium sulfate. The suitable crystals grew within 1–3 days to dimensions of approximately 200 × 50 × 10 μm. The apo *Ec*YjgB crystals were soaked into the crystallizing buffer supplemented with 5 mM NADP for 30 min. at 7 °C to enable the formation of the *Ec*YjgB-NADP complex. In this case, apo-form crystals were prepared within 1 day by hanging-drop vapor diffusion method at 20 °C, under the condition of 0.08 M Ammonium citrate tribasic pH 7.0 and 18% PEG 3350.

### 4.3. Data Collection and Processing

A reservoir solution containing 30% (*v*/*v*) glycerol was used as a cryo-protectant for the crystals. The data sets were collected at 100 K at 7A of the Pohang Accelerator Laboratory (Pohang, Korea). The best apo and NADP-complexed crystals were diffracted to resolutions of 1.55 and 1.45 Å, respectively. The apo crystals belonged to the *C2* space group with unit cell parameters of *a* = 133.14 Å, *b* = 64.47 Å, *c* = 81.66 Å, α = γ = 90.00°, and β = 106.14°, and NADP-complexed possessed *C222_1_* unit cell of *a* = 64.97 Å, *b* = 138.99 Å, *c* = 168.51 Å, and α = β = γ = 90.00°. The collected data were commonly indexed, integrated, and scaled while using the *HKL2000* suite [27]. Table 1 summarizes the statistics of the collected data.

### 4.4. Structure Determination and Refinement

The crystal structure was determined while using molecular replacement (MR) with the software *Phaser-MR* from *PHENIX* suite [28] using the crystal structure of sinapyl alcohol dehydrogenase from *Populus tremuloides* (PDB code: 1YQD) [17] as a search model. Model building was performed using the programs *ARP/wARP* [29] and *Wincoot* [30]. The refinement was performed using *phenix.refine* [31], and the final models of apo and complex had *R_work_* and *R_free_* values of 16.1% and 19.4%, and 17.7% and 22.1%, respectively. The geometric parameters of the final model were validated using *WinCoot* and *MolProbity* [32]. The experimental data of the structural models were assessed while using *SFCHECK* [33], and the refined models were deposited in the Protein Data Bank. Table 1 provides the refinement statistics.

### 4.5. Docking Simulation

The simulations of docking to the complex structure were performed using the *Autodock Vina* program [24]. The complex structure containing NADP and the fragment of PEG were used as a template. After removing the fragment of PEG, hydrogen atoms were added in accordance with only polar atoms. The structure of ligands were generated from the simplified molecular-input line-entry system and prepared as pdbqt files using *CCP4MG* (version 2.10.11, University of York, UK) [34] and *Autodocking tools* software (version 4.2, San Diego, CA, USA) [35]. The grid was set up with x = 20.52, y = 41.94, and z = 19.22 at center_x = 65.40, y = 59.95, and z = 100.22. An exhaustiveness value of eight was used for running software. The simulation results were checked while using *PyMOL* software (version 2.4, Schrödinger Inc., New York, USA).

## 5. Conclusions

In summary, we determined that the crystal structure of *E. coli* YjgB could be useful for designing a bio-synthetical pathway for the production of butanol, isobutanol, propanediol, butanediol, 2-PE, and 2-PEAc. We identified that a PEG fragment occupies the substrate binding pocket via π-π stackings by NADP binding. Through docking simulation for the complex structure using four substrates, we suggested that π-π stacking of aromatic rings in the active site is responsible for the broad substrate selectivity for several substrates.

## Figures and Tables

**Table 1:** Data collection, phasing, and refinement statistics for YjgB from *E. coli*.

	Apo	PEG-NADP Complex
Data collection		
Wavelength (Å)	0.97934	1.00003
Space group	*C2*	*C222_1_*
Cell dimensions		
*a*, *b*, *c* (Å)	133.14, 64.47, 81.66	64.97, 138.99, 168.51
α, β, γ (°)	90.00, 106.14, 90.00	90.00, 90.00, 90.00
Resolution (Å)	50.00–1.55 (1.58–1.55) ^a^	30.00–2.00 (2.06–2.00)
*R*_merge_ (%) ^b^	7.2 (57.3)	10.9 (59.2)
*I*/*σ* (*I*)	36.2 (3.8)	19.2 (5.3)
Unique reflection	95566 (4760)	51912 (4358)
Completeness (%)	100.0 (100.0)	99.9 (100.0)
Redundancy	7.4 (7.4)	13.8 (14.1)
CC_1/2_	97.5 (89.4)	99.9 (96.5)
Refinement		
Resolution (Å)	25.02–1.55	29.55–2.00
No. reflections	95547	99594
*R*_work_ (%) ^c^/*R*_free_ (%) ^d^	16.1/19.4	16.0/20.0
No. atoms	6093	5493
Zn	4	4
Glycerol	36	12
NO_3_	8	
NADP		96
PEG		14
Water	790	591
Averaged *B*-factors	24.8	27.9
R.m.s. deviations from ideal value		
Bond lengths (Å)	0.013	0.008
Bond angles (°)	1.400	0.903
PDB ID	7BU2	7BU3

^a^ Values in parentheses are for highest-resolution shell. ^b^ *R*_merge_ *= ∑_hkl_ ∑_i_|I_i_(hkl)* *− <I(hkl)>|/∑_hkl_ ∑_i_ I_i_(hkl)* where *<I(hkl)>* is the average intensity of the *i*-th observations. ^c^ *R*_work_ *= ∑_hkl_ ||F_obs_(hkl)|* *− |F_calc_(hkl)||/∑_hkl_ |F_obs_(hkl)|*. *R_work_* is calculated with 95% of reflections used for structure refinement. ^d^ *R*_free_ is calculated for the remaining 5% of reflections randomly selected and excluded from refinement.

**Table 2:** B-factor comparisons for Zn and its coordinate residues.

	Apo (Å^2^)	NADP-PEG Complex (Å^2^)	Ratio (Complex/Apo)
Zn	17.8	38.8	2.2
Cys41	17.0	31.9	1.9
His63	15.5	31.9	2.1
Glu64	14.6	33.8	2.3
Cys152	15.6	28.1	1.8
Whole protein	24.8	27.9	1.1
